# Strong and sustainable primary healthcare is associated with a lower risk of hospitalization in high risk patients

**DOI:** 10.1038/s41598-021-83962-y

**Published:** 2021-02-23

**Authors:** Olga A. Sawicki, Angelina Mueller, Renate Klaaßen-Mielke, Anastasiya Glushan, Ferdinand M. Gerlach, Martin Beyer, Michel Wensing, Kateryna Karimova

**Affiliations:** 1 Institute of General Practice, Goethe University, 60590 Frankfurt, Germany; 2 Institute of Medical Informatics, Biometry and Epidemiology, Ruhr University, 44789 Bochum, Germany; 3 Department of General Practice and Health Services Research and Implementation Science, 69129 Heidelberg, Germany

**Keywords:** Health care, Health services

## Abstract

In 2004, Germany introduced a program based on voluntary contracting to strengthen the role of general practice care in the healthcare system. Key components include structured management of chronic diseases, coordinated access to secondary care, data-driven quality improvement, computerized clinical decision-support, and capitation-based reimbursement. Our aim was to determine the long-term effects of this program on the risk of hospitalization of specific categories of high-risk patients. Based on insurance claims data, we conducted a longitudinal observational study from 2011 to 2018 in Baden-Wuerttemberg, Germany. Patients were assigned to one or more of four open cohorts (in 2011, elderly, n = 575,363; diabetes mellitus, n = 163,709; chronic heart failure, n = 82,513; coronary heart disease, n = 125,758). Adjusted for key patient characteristics, logistic regression models were used to compare the hospitalization risk of the enrolled patients (intervention group) with patients receiving usual primary care (control group). At the start of the study and throughout long-term follow-up, enrolled patients in the four cohorts had a lower risk of all-cause hospitalization and ambulatory, care-sensitive hospitalization. Among patients with chronic heart failure and coronary heart disease, the program was associated with significantly reduced risk of cardiovascular-related hospitalizations across the eight observed years. The effect of the program also increased over time. Over the longer term, the results indicate that strengthening primary care could be associated with a substantial reduction in hospital utilization among high-risk patients.

## Introduction

Strong primary care, defined as coordinated, comprehensive first-contact care^1^, is the cornerstone of the healthcare system and associated with a better functioning health system^2^. The benefits of well-developed primary care systems are confirmed. Systematic literature reviews reported on mortality reduction, improved population health outcomes, and lower healthcare costs associated with strong primary care^3–5^. Despite the great recognition of its impact and the importance to health systems^6^, there have been limited restructuring efforts focused on primary care services.

In Germany, the fragmentation of care, especially in the ambulatory care sector^7^, is a source of increasing concern. Registration with a general practitioner (GP) is not mandatory, and usual care entails no formal gatekeeping procedure. Fragmentation has led to poor continuity of care, redundant services, unnecessary risks, and higher costs^8^. In 2004, the primary healthcare system in Germany was reorganized through the creation of a legal framework aiming to strengthen primary care. In the federal state of Baden-Wuerttemberg, where the program was implemented, the GP is the gatekeeper responsible for the coordination of care. The program consists of structured management of chronic diseases, continuous data-based quality improvement using computerized decision support for prescribing medication, and payment mainly by capitation per enrolled patient^9^. The program was expected to improve long-term health outcomes^10–12^. Previous studies have shown the program to have beneficial effects in terms of hospitalization rates, cost, and 5-year mortality, in the total participant population^9,13^. Adverse clinical outcomes for patients with both diabetes mellitus and cardiac conditions also improved, compared to usual care^14,15^. Detailed insights into the reasons behind these effects are limited. As an analysis focusing on chronic disease suggests health service use among high-need patients tends to decline^15^, we hypothesized that the impact would be highest in patients at high risk of disease deterioration^10,16,17^. The aim of this study was to assess the long-term effects of the primary care program on hospitalization risk among high-risk patients over a considerably longer follow-up of eight years. Since the impact of demographic change on healthcare systems is a pressing issue in all developed countries^18^, it is widely recommended that the long-term effects of interventions in high-risk patients are studied before setting health policy.

## Results

### Characteristics of patients

In 2011, 638,452 patients were included and assigned to one or several of the four cohorts: elderly, diabetes, chronic heart failure (CHF), and/or coronary heart disease (CHD). Of those, 51.4% received care under the primary care program and 48.6% received usual care. Table 1 summarizes the number and percentage of included patients by year. Demographic and clinical characteristics of included patients by cohort are shown in Table 2. Patients enrolled in the primary care program had more comorbidities than non-enrolled patients, as measured using the Charlson index.

**Table 1:** Number and percentage of included patients.

Year under observation	Usual care, number (%)	Primary care program, number (%)	Overall, number
2011	310,029 (48.6)	328,423 (51.4)	638,452
2012	312,862 (46.9)	354,369 (53.1)	667,231
2013	300,131 (44.0)	382,097 (56.0)	682,228
2014	283,597 (41.4)	401,003 (58.6)	684,600
2015	282,049 (39.0)	440,661 (61.0)	722,710
2016	274,819 (38.0)	449,133 (62.0)	723,952
2017	269,538 (36.9)	461,465 (63.1)	731,003
2018	260,932 (35.8)	467,860 (64.2)	728,792

**Table 2:** Characteristics of patients by group in two of the final three quarters of 2011.

Characteristics	Elderly			Diabetes mellitus type 2			CHF			CHD		
	Control group	Intervention group	*P*-value	Control group	Intervention group	*P*-value	Control group	Intervention group	*P*-value	Control group	Intervention group	*P*-value
Number of patients	282,228	293,135		72,480	91,229		38,551	43,962		57,666	68,092	
**Socio-economic parameter**												
Mean age, years (SD)	76.3 (7.3)	75.7 (6.8)	> 0.001	71.5 (11.3)	70.8 (11.2)	> 0.001	78.2 (10.3)	77.1 (10.0)	> 0.001	74.3 (10.4)	73.7 (10.2)	> 0.001
Sex, % women	61.3	59.6	> 0.001	54.4	53.4	> 0.001	61.9	58.7	> 0.001	44.3	42.7	> 0.001
Need for nursing, %	17.4	12.7	> 0.001	15.6	11.8	> 0.001	30.0	23.3	> 0.001	16.4	13.1	> 0.001
Rural practice, %	48.5	46.0	> 0.001	49.9	47.7	> 0.001	45.6	44.0	> 0.001	50.7	48.0	> 0.001
Group practice*	36.0	47.7	> 0.001	38.1	48.5	> 0.001	36.3	46.6	> 0.001	37.5	47.6	> 0.001
**Health service utilization, %**												
DMP CHD	5.7	11.7	> 0.001	8.5	15.3	> 0.001	11.7	23.6	> 0.001	28.2	53.4	> 0.001
DMP DM	15.1	25.3	> 0.001	54.0	77.9	> 0.001	20.5	33.2	> 0.001	22.8	34.8	> 0.001
Hospital admission	8.4	7.9	> 0.001	9.9	9.2	> 0.001	15.8	15.0	> 0.001	15.0	14.1	> 0.001
Mean Charlson Comorbidity Index (SD)	2.1 (2.2)	2.2 (2.2)	> 0.001	3.3 (2.3)	3.4 (2.3)	> 0.001	3.6 (2.5)	3.7 (2.5)	> 0.001	3.0 (2.5)	3.1 (2.5)	> 0.001
**Comorbid condition, %**												
Renal failure	7.8	8.0	0.005	11.3	10.9	0.003	17.9	17.8	0.6	13.5	13.4	0.542
COPD	9.0	10.2	> 0.001	10.3	11.3	> 0.001	15.6	17.3	> 0.001	13.3	14.7	> 0.001
Dementia	7.8	5.7	> 0.001	5.8	4.5	> 0.001	10.3	8.2	> 0.001	6.3	5.1	> 0.001
Depression	15.1	14.3	> 0.001	16.5	15.6	> 0.001	19.3	17.6	> 0.001	17.2	15.6	> 0.001
**Cardiovascular history, %**												
CHD	16.9	19.1	0.001	24.0	24.8	> 0.001	38.7	41.8	> 0.001	100.0	100.0	n.a
CHF	12.3	13.4	> 0.001	17.0	16.9	0.834	100.0	100.0	n.a	25.9	27.0	> 0.001
Myocardial infarction	0.8	0.7	0.033	1.0	0.9	0.137	1.3	1.1	0.119	1.5	1.4	0.115
Hypertension	68.3	70.5	> 0.001	78.1	77.8	0.219	75.9	77.1	> 0.001	78.6	78.8	0.285
Atrial fibrillation	8.9	9.4	> 0.001	11.6	10.8	> 0.001	26.9	26.4	0.082	16.9	16.3	0.004

*CHF* chronic heart failure, *CHD* coronary heart disease, *COPD* chronic obstructive pulmonary disease, *DMP* disease management program, *DM* diabetes mellitus, *SD* standard deviation. *Doctors working in a group practice.

### Hospitalization in the elderly

**Figure 1:**
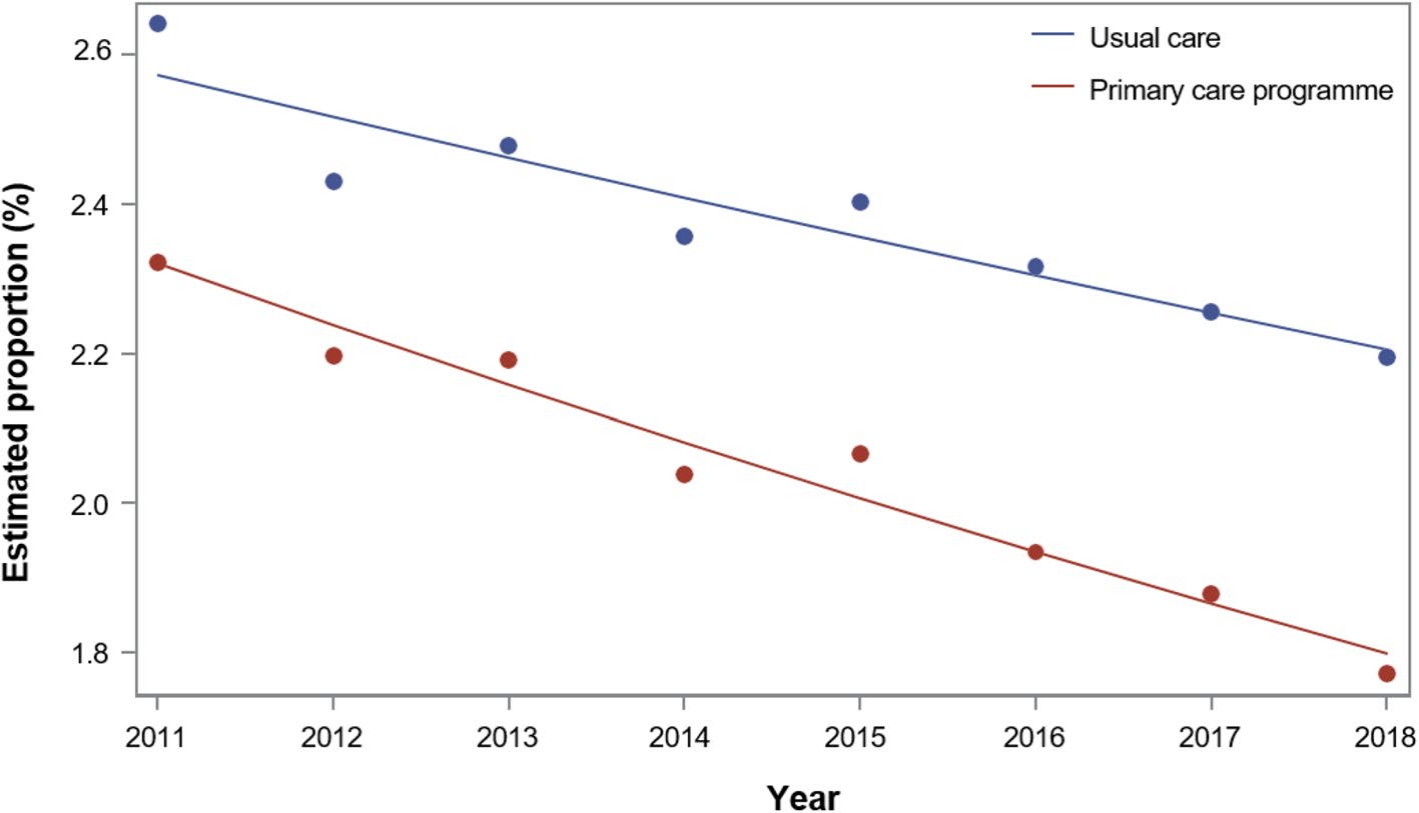
Estimated proportions of all-cause hospitalization in the elderly, based on generalized linear models of the 2011–2018 longitudinal analysis.

Table 3 presents the results of the longitudinal analysis. At the start of the study, elderly patients in the intervention group had a significantly lower risk of all-cause hospitalization [Odds Ratio (OR): 0.908; 95% CI: 0.900–0.916; Fig. 1] and ambulatory, care-sensitive hospitalizations (ACSH) (OR 0.900; 95% CI: 0.882–0.919; see Supplementary Fig. S1 online). Whereas the estimated proportions displayed a significant downward trend in the control group, the magnitude of the intervention effect increased throughout follow-up. Hip fracture-related hospitalization risk was significantly lower in the intervention group at the start of the study (OR: 0.943; 95% CI: 0.905–0.983), with no significant difference in the trend between groups over the eight-year period (see Supplementary Fig. S2 online). Older patients had increased risk of hospitalization as did patients with greater Charlson index score, and comorbidities such as renal failure, hypertension, COPD, dementia, and depression.

**Table 3:** Hospitalization per year and patient: Odds ratios* based on the longitudinal analysis (2011–2018).

	Intervention group versus control group (intervention effect) at the start of the study, OR (95% CI)	p-value	Control group per year (time trend), OR (95% CI)	*P*-value	Time trend in the intervention group versus time trend in the control group, OR (95% CI)	*P*-value
**Elderly**						
Hospitalization	0.908 (0.900–0.916)	< 0.0001	0.973 (0.971–0.974)	< 0.0001	0.981 (0.979–0.983)	< 0.0001
ACSH	0.900 (0.882–0.919)	< 0.0001	0.978 (0.974–0.981)	< 0.0001	0.986 (0.981–0.990)	< 0.0001
Hospitalization for hip fracture	0.943 (0.905–0.983)	0.0055	0.984 (0.977–0.991)	< 0.0001	0.992 (0.982–1.002)	0.1046
**Diabetes mellitus type 2**						
Hospitalization	0.949 (0.935–0.964)	< 0.0001	0.969 (0.966–0.971)	< 0.0001	0.983 (0.980–0.987)	< 0.0001
ACSH	0.943 (0.914–0.974)	0.0004	0.971 (0.966–0.976)	< 0.0001	0.984 (0.977–0.991)	< 0.0001
Diabetes-related hospitalization	0.833 (0.794–0.874)	< 0.0001	0.934 (0.926–0.942)	< 0.0001	0.996 (0.985–1.007)	0.4610
**Chronic heart failure**						
Hospitalization	0.980 (0.960–0.999)	0.0409	0.970 (0.966–0.973)	< 0.0001	0.975 (0.971–0.979)	< 0.0001
ACSH	0.978 (0.941–1.017)	0.2638	0.987 (0.980–0.994)	0.0001	0.970 (0.961–0.978)	< 0.0001
CVD-related hospitalization	0.925 (0.901–0.949)	< 0.0001	0.962 (0.957–0.966)	< 0.0001	0.975 (0.969–0.981)	< 0.0001
**Coronary heart disease**						
Hospitalization	0.962 (0.946–0.979)	< 0.0001	0.969 (0.966–0.972)	< 0.0001	0.983 (0.979–0.986)	< 0.0001
ACSH	0.970 (0.936–1.005)	0.0969	0.983 (0.977–0.989)	< 0.0001	0.984 (0.976–0.992)	< 0.0001
CVD-related hospitalization	0.921 (0.901–0.942)	< 0.0001	0.965 (0.961–0.969)	< 0.0001	0.978 (0.973–0.983)	< 0.0001

*P*-values relate to adjusted effect estimates in generalized linear models based on a longitudinal analysis from 2011 to 2018. ACSH, ambulatory, care-sensitive hospitalization.

*CVD* cardiovascular disease, *OR* odds ratio. *Odds ratios for all covariates and outcome measures are shown online in the Supplementary Table S2–13.

### Hospitalization in diabetics

**Figure 2:**
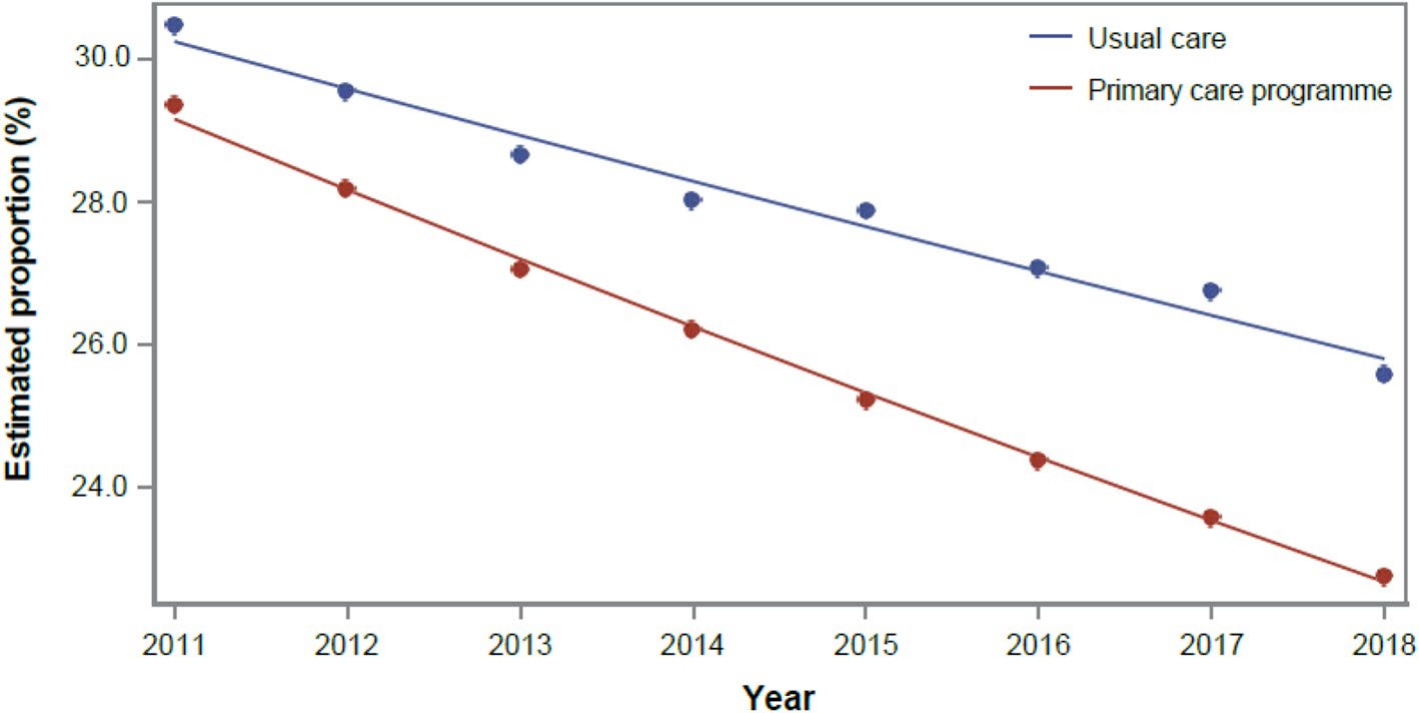
Estimated proportions of all-cause hospitalization in diabetics, based on generalized linear models of the 2011–2018 longitudinal analysis.

At the start of the study and throughout the long-term follow-up, enrolled patients with diabetes mellitus had a lower risk of all-cause hospitalization (OR 0.949; 95% CI: 0.935–0.964; Fig. 2) and ACSH (OR 0.943; 95% CI: 0.914–0.974; see Supplementary Fig. S3 online). A decreasing trend was observed in the control group over time. The effect of the program increased over time, while it remained stable for diabetes-related hospitalizations (see Supplementary Fig. S4 online). Factors associated with increased risk of hospitalization were previous hospitalization, greater Charlson index score as well as the presence of renal failure and hypertension.

### Hospitalization in patients with cardiovascular diseases

**Figure 3:**
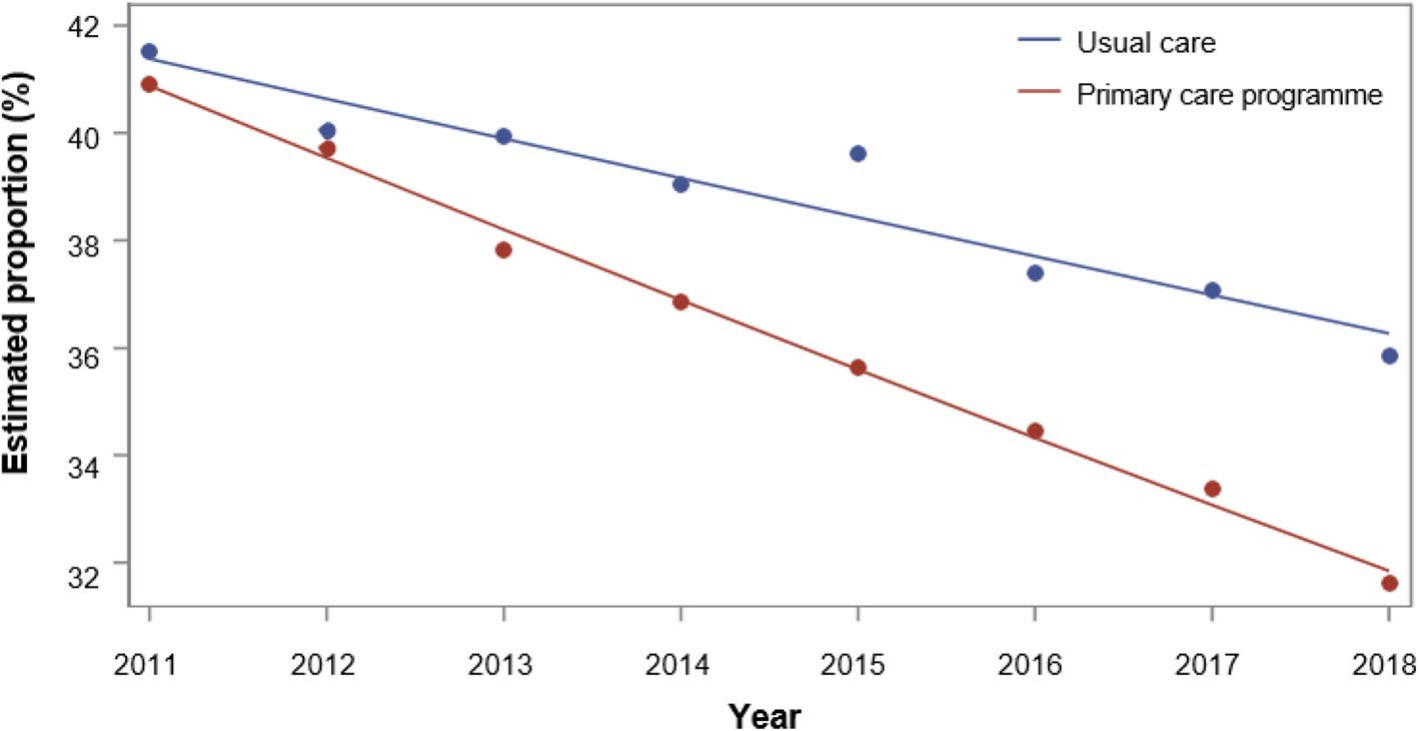
Estimated proportions of all-cause hospitalization in CHF patients, based on generalized linear models of the longitudinal analysis from 2011 to 2018.

**Figure 4:**
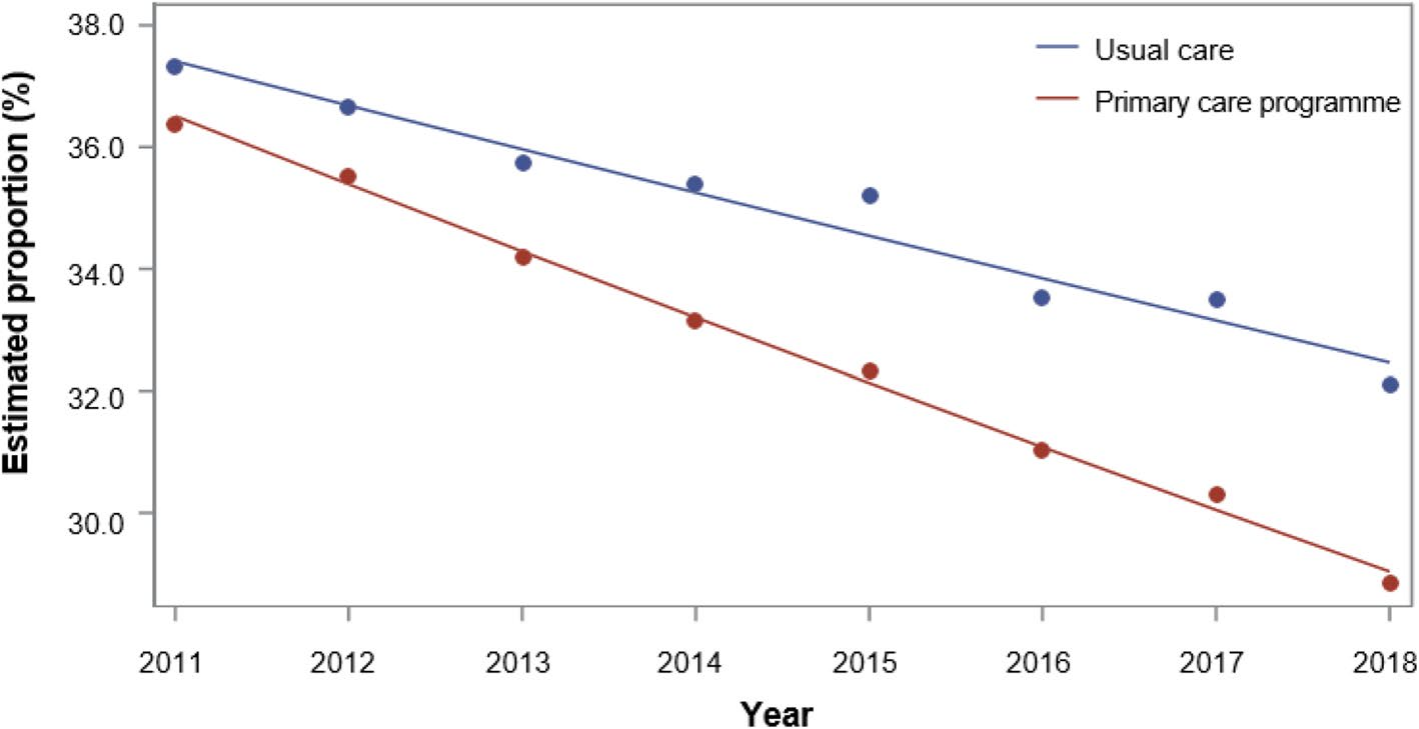
Estimated proportions of all-cause hospitalization in CHD patients, based on generalized linear models of the longitudinal analysis from 2011 to 2018.

At the start of the study, enrolled CHF and CHD patients had had significantly fewer all-cause (CHF OR 0.980; 95% CI: 0.960–0.999; CHD OR 0.962; 95% CI: 0.946–0.979; Figs. 3,4) and cardiovascular-related hospitalizations (CHF OR 0.925; 95% CI: 0.901–0.949; CHD OR 0.921; 95% CI: 0.901–0.942; see Supplementary Fig. S5–6 online). While the estimated proportion of inpatients followed a downward trend in the control group, the effect of the program increased. Among the two cardiovascular diseases cohorts, the intervention effect on ACSH was not significant at the start of the study but grew in magnitude over the eight-year period and became significant by the end (CHF OR 0.975; 95% CI: 0.969–0.981; CHD OR 0.978; 95% CI: 0.973–0.983; see Supplementary Fig. S7–8 online). Having a greater Charlson index score, renal failure, and hypertension was associated with a higher risk to be hospitalized; similarly to previous hospitalization. In contrast, participation in disease management program (DMP) for diabetes was protective against hospitalization.

## Discussion

This study of routine healthcare revealed a correlation between stronger primary care and a substantial decline in hospitalization risk among elderly multi-morbid patients. Both at the start of the study and throughout the follow-up, we found that high-risk patients that were enrolled in the program had a lower risk of hospitalization. Furthermore, although we observed a downward trend in the estimated proportions of inpatients in both intervention and control groups over the eight-year period, the intervention effect increased over the years, although not for hip-fracture and diabetic-related hospitalizations. It thus could be plausible that the increasing difference between the groups reflects the sustainable effect of the primary care program. The general downward trend observed in hospitalization may be the result of improved quality of care and the wider use of outpatient diagnostics and therapy^19^.

The impact is higher in high-risk patients than the overall population that was evaluated earlier on in the programme^20^. At baseline in 2011, the total participant population showed a 10.7% reduction in hospital admissions^20^. In our study, the risk of hospitalization during the same year was up to 16.4% lower among enrolled high-risk patients. Such a difference is likely to be important for both patients and healthcare providers. The long-term benefit of the program was greatest among CHF and CHD patients. The study finding that elevated risk of hospitalization among high-risk patients was consistently associated with greater Charlson index score, renal failure, and hypertension, has been described in previous studies^21–24^.

In elderly patients, the study showed that strong primary care could have a substantial and positive impact on hospitalization risk for ambulatory care-sensitive conditions (ACSC) but did not lead to any long-term decline in hip fracture-related hospitalization. Possible explanations are that the assessed patients were too healthy, or that comprehensive geriatric assessments in outpatient settings are ineffective. Numerous studies have indicated that the benefits of outpatient-based geriatric assessments are limited in comparison to hospital or rehabilitation unit models^25,26^. Nevertheless, improved rates of ACSC admissions in enrolled patients may be partially due to an emphasis on the core coordinating functions of primary care. The program delivers medical care that is based on recommended standards, and trained practice assistants work in concert with the GP and specialists to coordinate services across the entire care continuum. Moreover, in pursuit of judicious drug use and high-quality drug management, GPs regularly participate in pharmacotherapy peer-group training sessions, and use electronic decision support when prescribing medications.

In diabetes patients, an improvement in overall hospitalization risk was observed. However, the lack of any association between program- and diabetes-related hospitalization over time is worthy of note. It is possible that improvements in diabetes care are more difficult to achieve due to the overall high quality of usual care^27^. Diabetes has benefitted from DMP for more than two decades in southern Germany^28^, and the German diabetes-DMP has been particularly successful in implementing comprehensive care, which has also led to high participation rates outside the present programme^29^.

In CHF and CHD patients, the results suggest that strong primary care has considerable potential to reduce hospitalization risk. The impact of the program is remarkably high in comparison to usual care. Since hospital admissions for both cardiovascular diseases are sensitive to strong primary care, these findings may be explained by suboptimal usual care. As the DMP for CHF was initiated only one year ago, evidence on the effectiveness of this program is still lacking. Current adoption patterns of the DMP for CHD may have encouraged relatively weak implementation^30^. Overall, our study implied that strong primary care appears to be associated with a substantial reduction in the hospital utilization of high-risk patients over the longer term. The program delivered structured management of chronic diseases, and provided additional incentives for specially trained practice assistants to help coordinate care, as it might have improved the quality of treatment and clinical outcomes of patients^31^. Furthermore, as participating GPs met specific quality requirements, e.g. they based necessary adjustments on a data-driven improvement system, used computerized clinical decision-support, and regularly participated in peer-group training sessions, they probably had greater expertise than those providing usual care. Although our results do not enable the identification of a key factor responsible for reducing hospitalization, the primary care structure in southern Germany permits the delivery of high-quality chronic care. In the United States, patient-centered medical homes have been identified as a potential means of supporting primary care delivery^5,32^. The patient-centered medical home has many elements of strong primary care and its implementation is associated with improved chronic disease care^33^, as well as reduced hospital and emergency department use^34^. Our results in a healthcare system that faces similar challenges to that in the United States support these findings.

The study has strengths and limitations. The evaluation of the sustained program took place in a real-world healthcare setting. The study benefitted from a large cross-sectoral dataset, and advanced statistical modelling. The follow-up period was also considerably longer than in any prior study evaluating the primary care program in southern Germany. Furthermore, we assessed the effect on hospitalization of different categories of high-risk patients. Another strength of the study is the comparison with control groups receiving usual care, and not simply with patients that had not enrolled in the program. The samples were therefore highly representative of patients that actually require healthcare.

The study also has limitations in terms of data analysis and selection bias. Like many studies based on secondary data analysis, we were reliant on data quality (e.g., correct coding), and in our case insurance claims data. Clinical information (e.g., diagnostic and prognostic information such as tobacco use) and social economic data were not available for adjustment purposes, which may have led to some residual confounding. Moreover, our methodical approach together with the lack of randomization excludes causal inference and therefore our results need to be interpreted with caution. When the program began, the proportion of hospitalized patients in the intervention groups was lower than in the control group. This may have led to an over-estimation of the program’s effect. Finally, since participation in the program is voluntary, it may have been influenced by patient and physician self-selection, as GPs and patients that chose to participate in the program voluntarily may have been more likely to take part in structured management programs, and receive guideline-directed therapies. Hence, it cannot be ruled out that the GPs bore greater responsibility for the identified effects than the program. Randomized controlled trials evaluating this program are needed. As fragmentation in the delivery of care accelerates, strengthening primary care would appear to be a viable means of reducing hospital utilization in elderly patients with multiple chronic conditions. Although implementation can be challenging, primary care should be strengthened in many other health systems.

## Methods

### Study design

The study was based on claims data. We used a comparative observational design with a follow-up of eight years. Open cohorts were analyzed, which means that all eligible patients of a given year were considered, so that the composition of the cohorts changed over the years and repeated measurement per subject was possible. Patients enrolled in the primary care program (intervention group) were compared with patients that were receiving usual care from a GP that did not participate in it (control group). Both groups were from the same geographical area in Germany. Baden-Wuerttemberg is a state in southwest Germany with about 11 million inhabitants and high participation in the primary care program. Analyses were based on routinely collected health data drawn from the administrative databases of the healthcare fund ‘Allgemeine Ortskrankenkasse’ (AOK) Baden-Wuerttemberg. AOK Baden-Wuerttemberg is the largest regional statutory health fund with > 40% coverage of the population. In 2017, more than 4000 GPs and 1.5 million patients were enrolled in the programme^35^. The analysis was carried out as part of a broader evaluation of the program. The study followed the STROBE (Strengthening the Reporting of Observational Studies in Epidemiology) guideline^36^ and the German standard for secondary data analysis (STROSA)^37^. The ethics committee of Frankfurt University Hospital (No. 470/13) approved the study.

### Study population

Patients were eligible to participate in the study, if they were insured by AOK in the year under observation (see Supplementary Fig. S9 online). Patients were excluded according to following predefined exclusion criteria: did not visit a GP, not continuously insured by AOK, < 18 years old, or participated in other healthcare programs, with the exception of nationwide DMPs for patients with chronic conditions. Participation in DMPs for diabetes, asthma, chronic obstructive pulmonary disease, and CHD were encouraged. In addition, we excluded patients if they did not participate in the program although their GP was participating, and participating patients with a non-participating GP. In the year under observation, patients were excluded if they or their GP switched groups, they changed their participating GP, they did not live in Baden-Wuerttemberg, or their GP practice was not located in Baden-Wuerttemberg. All inclusion and exclusion criteria were applied annually. In total, 3,056,472 patients were excluded of the study in 2011. Patients were assigned to the following disease cohorts based on the ICD codes of their diagnoses: diabetes mellitus type 2 (code E11), chronic heart failure (code I50), and coronary heart disease (codes I20-I25). Patients aged ≥ 65 years were assigned to the elderly cohort. The cohorts were not mutually exclusive. Patients in the intervention group had to be enrolled in the program and to provide written informed consent. Patients in the control group received usual primary care.

### Primary care program

The primary care program is a structured, coordinated, and comprehensive program. It seeks to improve care by strengthening the role of primary care. In comparison to other programs, which often focus on single-disease management or high-risk patients, the primary care program takes a population-based approach. It was arranged as a contract developed by health insurers in collaboration with GPs. Enrolment in the program is voluntary for both patients and GPs. The key elements of the program are structured management of chronic diseases, encouragement to participate in nationwide DMPs, coordinated access to other medical specialists, continuous data-oriented quality improvement and peer-group trainings, computerized decision support in prescribing medication, capitation-based reimbursement, and financial incentives to monitor patients with multiple chronic diseases. Since medication use is one of the risk factors for falls that is easy to modify, the program’s computerized decision support in prescribing medication would appear to be a promising approach to lowering the risk of falls among the elderly^38^. Participating patients in the program had to select a participating GP for a fixed period of time, and to accept that medical specialist care was only available upon referral (except for emergencies, gynecologists, and ophthalmologists). Thus, specialist care was usually only provided to participants after referral by a GP. Participating patients benefit from shorter waiting times, less out-of-pocket expenditure for medications, and evening appointments. A detailed description of the program’s rationale and implementation have been reported elsewhere^14,20^.

### Measures

All data were recorded annually. The main outcome measure in this study was at least one hospitalization, specified as a dichotomous indicator. Hospitalization was defined as any hospital admission, including rehospitalization, that included at least one overnight stay in the year under observation. Hospitalizations were classified as all-cause, for ambulatory care-sensitive conditions, and subgroup-specific (three pre-defined subgroups for each cohort). We assessed hip fracture-related hospitalization as a subgroup-specific outcome in the elderly. Among the elderly, most hip fractures result from falls^39^, and they are a leading cause of hospitalisation^38^. Among diabetics, we specifically assessed diabetes-related hospitalization, and among CHF and CAD patients, hospitalization for cardiovascular disease. The following parameters were annually documented (see Supplementary Table S1 online) and used for adjustment: patient age, gender, nursing care needs, rurality, group practice, health service utilization, and comorbid conditions. Comorbidity was also expressed using the Charlson index^40^. Included variables were medically justified and based on availability in the administrative data. The outcome measures we used were similarly defined, based on previous research^20^.

### Data-analysis

The characteristics of the participants at baseline are presented descriptively; Pearson's chi-square test and t-test were performed to detect differences between intervention and control group at the start of the study (Table 2).

To analyze the intervention effect, we used logistic regression models, adjusted for predetermined potential confounders (see Supplementary Table S1–13 online). Generalized estimation equations (GEE) were used to estimate the parameters of the model, taking into account inherent correlations in the subject caused by repeated measurement per subject. For the correlation we implied an autoregressive covariance structure. Odds ratios are calculated for the intervention group compared to the control group (intervention effect) at the beginning of the study and developments over time per year (time trend) in the control group. Additionally, an interaction between intervention and time is included in the model. The interaction parameter provides information on whether the intervention effect increases or decreases over time. Thus, the odds ratio for the interaction parameter can be interpreted as the factor by which the trend in the intervention group increases or decreases compared to the trend in the control group. In addition to the odds ratios, confidence intervals and p-values were reported. The results of the model calculations are visualized in figures by displaying the proportions predicted by the model as a function of time. SAS version 9.4 and IBM SPSS Statistics (version 25) were used for statistical analysis. Figures were obtained using SAS version 9.4 (SAS Institute Inc., Cary, North Carolina, USA).

## Supplementary Information

Supplementary Information

## Data Availability

AOK Baden-Wuerttemberg can be contacted for secondary analyses of their administrative data.
