# Supplementary material for: Strong and sustainable primary healthcare is associated with a lower risk of hospitalization in high risk patients
Source: Sci Rep. 2021 Feb 23;11:4349. doi: 10.1038/s41598-021-83962-y (PMC7902818; doi:10.1038/s41598-021-83962-y)
Supplement: Supplementary file 1 — Supplementary Information [file 41598_2021_83962_MOESM1_ESM.pdf]

Strong and sustainable primary healthcare is associated with a lower risk of hospitalization in high risk patients

Olga A. Sawicki[1,*], Angelina Mueller[1], Renate Klaaßen-Mielke[2], Anastasiya Glushan[1], Ferdinand M. Gerlach[1], Martin Beyer[1], Michel Wensing[3], and Kateryna Karimova[1]

[1]Institute of General Practice, Goethe University, Frankfurt, 60590, Germany
[2]Institute of Medical Informatics, Biometry and Epidemiology, Ruhr University, Bochum, 44789, Germany

[3]Department of General Practice and Health Services Research and Implementation Science, 69129, Heidelberg, Germany

[*]Corresponding author. Tel: + 49 69 6301 6476, Fax: + 49 69 6301 6428, E-mail address: sawicki@allgemeinmedizin.uni-frankfurt.de

Supplementary Table S1. Definition of covariates

CHD, coronary heart disease; CHF, chronic heart failure; COPD, chronic obstructive pulmonary disease; CVD, cardiovascular disease; DMP, disease management program; DM, diabetes mellitus.

Covariate	Operationalisation
Age	Age in decades in the year of observation
Sex	Male or female
Need for nursing	Registered need for nursing care or no need of nursing care in the year of observation
Rural practice	Yes or no (according to zip code and area classification of the Federal Institute for Research on Building, Urban Affairs and Spatial Development)
Group practice	No or yes; Practising in a group practice with more than one doctor
DMP CHD	Yes or no; Participation in disease management program for CHD (used for the CHD and CHF cohort)
DMP DM	Yes or no; Participation in disease management program for diabetes mellitus typ 1 or 2 (used only for the diabetes, CHD and CHF cohort)
Hospital admission	Yes or no. Hospital admission for any condition during the previous year
Charlson Comorbidity Index	0-37; Calculated according to Sundararajan et al.[25]
Renal failure	ICD-10: N18.2, N18.3, N18.4, N18.5 , N18.8, N18.9 at least two outpatient diagnoses, or one inpatient diagnosis in the year under observation
COPD	ICD-10: J44 at least two outpatient diagnoses, or one inpatient diagnosis in the year under observation
Dementia	ICD-10: F00, F01, F02, F03, G30 at least two outpatient diagnoses, or one inpatient diagnosis in the year under observation (used only for the elderly cohort)
Depression	ICD-10: F32, F33 at least two outpatient diagnoses, or one inpatient diagnosis in the year under observation (used only for the elderly cohort)
CHD	ICD-10: I25.0, I25.1, I25.2, I25.5, I25.6, I25.8, I25.9 at least two outpatient diagnoses, or one inpatient diagnosis during the previous year
CHF	ICD-10: I50 at least two outpatient diagnoses, or one inpatient diagnosis during the previous year
Myocardial infarction	ICD-10: I21, I22, I25.2 as main inpatient diagnosis in the year under observation, or one inpatient diagnosis during the previous year
Hypertension	ICD-10: I10-I15 at least two outpatient diagnoses, or one inpatient diagnosis in the year under observation
Atrial fibrillation	ICD-10: I48.0, I48.1, I48.2, I48.3, I48.4, I48.9 at least two outpatient diagnoses, or one inpatient diagnosis during the previous year

Supplementary Table S2. Odds ratios for all-cause hospitalization in the elderly, based on generalized linear models of the longitudinal analysis from 2011-2018
CHD, coronary heart disease; CHF, chronic heart failure; COPD, chronic obstructive pulmonary disease.

**Variable**	**Interpretation**	**Odds Ratio**	**Lower 95% CI**	**Upper 95% CI**	**p-value**
Intervention effect at the start of the study	Primary care program versus control group	0.908	0.900	0.916	<0.0001
Time trend in the unexposed group	Per year	0.973	0.971	0.974	<0.0001
Time trend modified by intervention	Primary care program versus control group per year	0.981	0.979	0.983	<0.0001
Age	In decades	1.073	1.069	1.078	<0.0001
Sex	Male versus female	1.042	1.036	1.048	<0.0001
Group practice	Yes versus no	1.001	0.995	1.006	0.7427
Rural practice	No versus yes	0.985	0.979	0.990	<0.0001
Charlson Comorbidity Index	Per point	1.354	1.352	1.356	<0.0001
Need for nursing	Yes versus no	0.638	0.633	0.643	<0.0001
Renal failure	Yes versus no	1.467	1.455	1.480	<0.0001
CHF	Yes versus no	0.855	0.849	0.862	<0.0001
Hypertension	Yes versus no	1.486	1.477	1.496	<0.0001
COPD	Yes versus no	1.159	1.149	1.169	<0.0001
Atrial fibrillation	Yes versus no	1.233	1.222	1.243	<0.0001
Myocardial infarction one inpatient diagnosis in the previous year	Yes versus no	1.137	1.110	1.164	<0.0001
Depression	Yes versus no	1.233	1.224	1.241	<0.0001
CHD	Yes versus no	0.962	0.956	0.969	<0.0001
Dementia	Yes versus no	1.311	1.298	1.324	<0.0001
Hospital admission	Yes versus no	0.911	0.905	0.916	<0.0001

Supplementary Table S3. Odds ratios for hospitalizations for ambulatory care-sensitive conditions in the elderly, based on generalized linear models of the longitudinal analysis from 2011-2018
CHD, coronary heart disease; CHF, chronic heart failure; COPD, chronic obstructive pulmonary disease.

Variable	Interpretation	Odds Ratio	Lower 95% CI	Upper 95% CI	p-value
Intervention effect at the start of the study	Primary care program versus control group	0.900	0.882	0.919	<0.0001
Time trend in the unexposed group	Per year	0.978	0.974	0.981	<0.0001
Time trend modified by intervention	Primary care program versus control group per year	0.986	0.981	0.990	<0.0001
Age	In decades	1.084	1.074	1.094	<0.0001
Sex	Male versus female	0.942	0.929	0.954	<0.0001
Group practice	Yes versus no	0.991	0.980	1.003	0.1580
Rural practice	No versus yes	1.024	1.012	1.036	0.0001
Charlson Comorbidity Index	Per point	1.200	1.198	1.203	<0.0001
Need for nursing	Yes versus no	1.793	1.765	1.820	<0.0001
Renal failure	Yes versus no	1.580	1.557	1.603	<0.0001
CHF	Yes versus no	0.960	0.946	0.974	<0.0001
Hypertension	Yes versus no	1.628	1.603	1.655	<0.0001
COPD	Yes versus no	2.304	2.270	2.339	<0.0001
Atrial fibrillation	Yes versus no	0.998	0.982	1.014	0.7949
Myocardial infarction one inpatient diagnosis in the previous year	Yes versus no	1.094	1.050	1.140	<0.0001
Myocardial infarction as main inpatient diagnosis	Yes versus no	1.157	1.110	1.207	<0.0001
Depression	Yes versus no	1.236	1.218	1.253	<0.0001
CHD	Yes versus no	0.989	0.975	1.002	0.1070
Dementia	Yes versus no	1.383	1.360	1.407	<0.0001
Hospital admission	Yes versus no	1.463	1.445	1.481	<0.0001

Supplementary Table S4. Odds ratios for hospitalization for hip fracture in the elderly, based on generalized linear models of the longitudinal analysis from 2011-2018

CHD, coronary heart disease; CHF, chronic heart failure; COPD, chronic obstructive pulmonary disease.

**Variable**	**Interpretation**	**Odds Ratio**	**Lower 95% CI**	**Upper 95% CI**	**p-value**
Intervention effect at the start of the study	Primary care program versus control group	0.943	0.905	0.983	0.0055
Time trend in the unexposed group	Per year	0.984	0.977	0.991	<0.0001
Time trend modified by intervention	Primary care program versus control group per year	0.992	0.982	1.002	0.1046
Age	In decades	2.093	2.057	2.130	<0.0001
Sex	Male versus female	0.646	0.629	0.664	<0.0001
Group practice	Yes versus no	0.978	0.956	1.001	0.0578
Rural practice	No versus yes	1.009	0.987	1.032	0.4268
Charlson Comorbidity Index	Per point	1.092	1.086	1.097	<0.0001
Need for nursing	Yes versus no	2.043	1.980	2.109	<0.0001
Renal failure	Yes versus no	1.659	1.610	1.709	<0.0001
CHF	Yes versus no	0.845	0.822	0.870	<0.0001
Hypertension	Yes versus no	1.203	1.169	1.238	<0.0001
COPD	Yes versus no	1.073	1.038	1.111	<0.0001
Atrial fibrillation	Yes versus no	1.027	0.996	1.059	0.0843
Myocardial infarction one inpatient diagnosis in the previous year	Yes versus no	0.865	0.772	0.968	0.0116
Myocardial infarction as main inpatient diagnosis	Yes versus no	0.882	0.792	0.982	0.0217
Depression	Yes versus no	1.130	1.100	1.161	<0.0001
CHD	Yes versus no	0.851	0.828	0.875	<0.0001
Dementia	Yes versus no	2.079	2.021	2.139	<0.0001
Hospital admission	Yes versus no	1.113	1.086	1.141	<0.0001

Supplementary Table S5. Odds ratios for all-cause hospitalization in diabetics, based on generalized linear models of the longitudinal analysis from 2011-2018

CHD, coronary heart disease; CHF, chronic heart failure; COPD, chronic obstructive pulmonary disease; DMP, disease management program; DM, diabetes mellitus.

Variable	Interpretation	Odds Ratio	Lower 95% CI	Upper 95% CI	p-value
Intervention effect at the start of the study	Primary care program versus control group	0.949	0.935	0.964	<0.0001
Time trend in the unexposed group	Per year	0.969	0.966	0.971	<0.0001
Time trend modified by intervention	Primary care program versus control group per year	0.983	0.980	0.987	<0.0001
Age	In decades	0.962	0.958	0.967	<0.0001
Sex	Male versus female	0.993	0.984	1.002	0.1203
Group practice	Yes versus no	1.010	1.002	1.019	0.0196
DMP DM	Yes versus no	0.732	0.726	0.739	<0.0001
Rural practice	No versus yes	0.996	0.988	1.005	0.4065
Charlson Comorbidity Index	Per point	1.380	1.377	1.384	<0.0001
Need for nursing	Yes versus no	1.654	1.635	1.674	<0.0001
Renal failure	Yes versus no	1.356	1.340	1.372	<0.0001
CHF	Yes versus no	0.903	0.893	0.913	<0.0001
Hypertension	Yes versus no	1.470	1.454	1.487	<0.0001
COPD	Yes versus no	1.118	1.103	1.132	<0.0001
Atrial fibrillation	Yes versus no	1.234	1.219	1.250	<0.0001
Myocardial infarction, one inpatient diagnosis during the previous year	Yes versus no	1.085	1.052	1.120	<0.0001
CHD	Yes versus no	1.046	1.035	1.056	<0.0001
Hospital admission	Yes versus no	1.305	1.294	1.317	<0.0001

Supplementary Table S6. Odds ratios for hospitalizations for ambulatory care-sensitive conditions in diabetics, based on generalized linear models of the longitudinal analysis from 2011-2018
CHD, coronary heart disease; CHF, chronic heart failure; COPD, chronic obstructive pulmonary disease; DMP, disease management program; DM, diabetes mellitus.

**Variable**	**Interpretation**	**Odds Ratio**	**Lower 95% CI**	**Upper 95% CI**	**p-value**
Intervention effect at the start of the study	Primary care program versus control group	0.943	0.914	0.974	0.0004
Time trend in the unexposed group	Per year	0.971	0.966	0.976	<0.0001
Time trend modified by intervention	Primary care program versus control group per year	0.984	0.977	0.991	<0.0001
Age	In decades	0.980	0.971	0.989	<0.0001
Sex	Male versus female	0.964	0.946	0.982	<0.0001
Group practice	Yes versus no	1.009	0.991	1.026	0.3225
DMP DM	Yes versus no	0.828	0.812	0.844	<0.0001
Rural practice	No versus yes	1.017	1.000	1.035	0.0530
Charlson Comorbidity Index	Per point	1.205	1.201	1.209	<0.0001
Need for nursing	Yes versus no	1.851	1.813	1.890	<0.0001
Renal failure	Yes versus no	1.699	1.665	1.734	<0.0001
CHF	Yes versus no	1.011	0.991	1.032	0.2806
Hypertension	Yes versus no	1.537	1.498	1.576	<0.0001
COPD	Yes versus no	1.914	1.873	1.955	<0.0001
Atrial fibrillation	Yes versus no	1.004	0.982	1.027	0.7330
Myocardial infarction, one inpatient diagnosis during the previous year	Yes versus no	1.041	0.990	1.095	0.1146
Myocardial infarction as main inpatient diagnosis	Yes versus no	1.138	1.076	1.203	<0.0001
CHD	Yes versus no	1.010	0.990	1.029	0.3404
Hospital admission	Yes versus no	1.593	1.566	1.621	<0.0001

Supplementary Table S7. Odds ratios for diabetic-related hospital admissions in diabetics, based on generalized linear models of the longitudinal analysis from 2011-2018

CHD, coronary heart disease; CHF, chronic heart failure; COPD, chronic obstructive pulmonary disease; DMP, disease management program; DM, diabetes mellitus.

Variable	Interpretation	Odds Ratio	Lower 95% CI	Upper 95% CI	p-value
Intervention effect at the start of the study	Primary care program versus control group	0.833	0.794	0.874	<0.0001
Time trend in the unexposed group	Per year	0.934	0.926	0.942	<0.0001
Time trend modified by intervention	Primary care program versus control group per year	0.996	0.985	1.007	0.4610
Age	In decades	0.747	0.737	0.758	<0.0001
Sex	Male versus female	1.183	1.151	1.217	<0.0001
Group practice	Yes versus no	1.004	0.978	1.032	0.7444
DMP DM	Yes versus no	1.128	1.095	1.163	<0.0001
Rural practice	No versus yes	0.959	0.933	0.985	0.0025
Charlson Comorbidity Index	Per point	1.223	1.217	1.229	<0.0001
Need for nursing	Yes versus no	1.871	1.812	1.933	<0.0001
Renal failure	Yes versus no	2.117	2.050	2.185	<0.0001
CHF	Yes versus no	0.987	0.956	1.019	0.4224
Hypertension	Yes versus no	1.613	1.550	1.679	<0.0001
COPD	Yes versus no	0.807	0.778	0.838	<0.0001
Atrial fibrillation	Yes versus no	0.962	0.928	0.998	0.0372
Myocardial infarction, one inpatient diagnosis during the previous year	Yes versus no	0.992	0.920	1.070	0.8403
Myocardial infarction as main inpatient diagnosis	Yes versus no	1.041	0.959	1.131	0.3366
CHD	Yes versus no	1.004	0.974	1.035	0.8107
Hospital admission	Yes versus no	1.557	1.515	1.599	<0.0001

Supplementary Table S8. Odds ratios for all-cause hospitalization in CHF patients, based on generalized linear models of the longitudinal analysis from 2011-2018
CHD, coronary heart disease; CHF, chronic heart failure; COPD, chronic obstructive pulmonary disease; DMP, disease management program; DM, diabetes mellitus.

Variable	Interpretation	Odds Ratio	Lower 95% CI	Upper 95% CI	p-value
Intervention effect at the start of the study	Primary care program versus control group	0.980	0.960	0.999	0.0409
Time trend in the unexposed group	Per year	0.970	0.966	0.973	<0.0001
Time trend modified by intervention	Primary care program versus control group per year	0.975	0.971	0.979	<0.0001
Age	In decades	0.954	0.949	0.959	<0.0001
Sex	Male versus female	1.028	1.016	1.040	<0.0001
Group practice	Yes versus no	1.012	1.001	1.022	0.0341
DMP DM	Yes versus no	0.572	0.564	0.579	<0.0001
DMP CHD	Yes versus no	0.996	0.980	1.011	0.5854
Rural practice	No versus yes	1.007	0.996	1.018	0.1861
Charlson Comorbidity Index	Per point	1.331	1.327	1.334	<0.0001
Need for nursing	Yes versus no	1.567	1.547	1.587	<0.0001
Renal failure	Yes versus no	1.651	1.628	1.673	<0.0001
Hypertension	Yes versus no	1.550	1.529	1.571	<0.0001
COPD	Yes versus no	1.114	1.099	1.130	<0.0001
Atrial fibrillation	Yes versus no	1.265	1.250	1.280	<0.0001
Myocardial infarction, one inpatient diagnosis during the previous year	Yes versus no	1.185	1.152	1.219	<0.0001
CHD	Yes versus no	1.001	0.988	1.014	0.8920
Hospital admission	Yes versus no	1.480	1.464	1.496	<0.0001

Supplementary Table S9. Odds ratios for hospitalizations for ambulatory care-sensitive conditions in CHF patients, based on generalized linear models of the longitudinal analysis from 2011-2018
CHD, coronary heart disease; CHF, chronic heart failure; COPD, chronic obstructive pulmonary disease; DMP, disease management program; DM, diabetes mellitus.

Variable	Interpretation	Odds Ratio	Lower 95% CI	Upper 95% CI	p-value
Intervention effect at the start of the study	Primary care program versus control group	0.978	0.941	1.017	0.2638
Time trend in the unexposed group	Per year	0.987	0.980	0.994	0.0001
Time trend modified by intervention	Primary care program versus control group per year	0.970	0.961	0.978	<0.0001
Age	In decades	0.970	0.959	0.981	<0.0001
Sex	Male versus female	0.984	0.962	1.006	0.1508
Group practice	Yes versus no	0.995	0.975	1.016	0.6334
DMP DM	Yes versus no	0.929	0.908	0.951	<0.0001
DMP CHD	Yes versus no	0.849	0.824	0.873	<0.0001
Rural practice	No versus yes	1.017	0.996	1.038	0.1076
Charlson Comorbidity Index	Per point	1.170	1.165	1.175	<0.0001
Need for nursing	Yes versus no	1.841	1.800	1.884	<0.0001
Renal failure	Yes versus no	1.654	1.617	1.692	<0.0001
Hypertension	Yes versus no	1.402	1.363	1.442	<0.0001
COPD	Yes versus no	2.184	2.135	2.234	<0.0001
Atrial fibrillation	Yes versus no	0.963	0.942	0.984	0.0006
Myocardial infarction, one inpatient diagnosis during the previous year	Yes versus no	1.047	0.998	1.097	0.0590
Myocardial infarction as main inpatient diagnosis	Yes versus no	1.161	1.088	1.239	<0.0001
CHD	Yes versus no	1.007	0.983	1.032	0.5600
Hospital admission	Yes versus no	1.596	1.564	1.629	<0.0001

Supplementary Table S10. Odds ratios for cardiovascular-related hospital admissions n in CHF patients, based on generalized linear models of the longitudinal analysis from 2011-2018

CHD, coronary heart disease; CHF, chronic heart failure; COPD, chronic obstructive pulmonary disease; DMP, disease management program; DM, diabetes mellitus.

Variable	Interpretation	Odds Ratio	Lower 95% CI	Upper 95% CI	p-value
Intervention effect at the start of the study	Primary care program versus control group	0.925	0.901	0.949	<0.0001
Time trend in the unexposed group	Per year	0.962	0.957	0.966	<0.0001
Time trend modified by intervention	Primary care program versus control group per year	0.975	0.969	0.981	<0.0001
Age	In decades	0.991	0.983	0.999	0.0276
Sex	Male versus female	1.024	1.009	1.040	0.0018
Group practice	Yes versus no	1.011	0.997	1.025	0.1411
DMP DM	Yes versus no	0.678	0.667	0.690	<0.0001
DMP CHD	Yes versus no	1.212	1.189	1.236	<0.0001
Rural practice	No versus yes	1.008	0.994	1.022	0.2820
Charlson Comorbidity Index	Per point	1.222	1.218	1.226	<0.0001
Need for nursing	Yes versus no	1.059	1.042	1.077	<0.0001
Renal failure	Yes versus no	1.899	1.870	1.929	<0.0001
Hypertension	Yes versus no	1.456	1.429	1.483	<0.0001
COPD	Yes versus no	1.020	1.003	1.038	0.0224
Atrial fibrillation	Yes versus no	1.533	1.511	1.556	<0.0001
Myocardial infarction, one inpatient diagnosis during the previous year	Yes versus no	1.520	1.472	1.569	<0.0001
CHD	Yes versus no	1.234	1.213	1.255	<0.0001
Hospital admission	Yes versus no	1.340	1.322	1.359	<0.0001

Supplementary Table S11. Odds ratios for all-cause hospitalization in CHD patients, based on generalized linear models of the longitudinal analysis from 2011-2018

CHD, coronary heart disease; CHF, chronic heart failure; COPD, chronic obstructive pulmonary disease; DMP, disease management program; DM, diabetes mellitus.

Variable	Interpretation	Odds Ratio	Lower 95% CI	Upper 95% CI	p-value
Intervention effect at the start of the study	Primary care program versus control group	0.962	0.946	0.979	<0.0001
Time trend in the unexposed group	Per year	0.969	0.966	0.972	<0.0001
Time trend modified by intervention	Primary care program versus control group per year	0.983	0.979	0.986	<0.0001
Age	In decades	0.966	0.960	0.971	<0.0001
Sex	Male versus female	0.971	0.961	0.981	<0.0001
Group practice	Yes versus no	1.017	1.007	1.027	0.0009
DMP DM	Yes versus no	0.565	0.558	0.572	<0.0001
DMP CHD	Yes versus no	0.947	0.937	0.957	<0.0001
Rural practice	No versus yes	0.997	0.987	1.007	0.5774
Charlson Comorbidity Index	Per point	1.368	1.365	1.372	<0.0001
Need for nursing	Yes versus no	1.555	1.535	1.577	<0.0001
Renal failure	Yes versus no	1.474	1.455	1.494	<0.0001
CHF	Yes versus no	0.895	0.886	0.904	<0.0001
Hypertension	Yes versus no	1.766	1.743	1.789	<0.0001
COPD	Yes versus no	1.078	1.064	1.093	<0.0001
Atrial fibrillation	Yes versus no	1.250	1.235	1.266	<0.0001
Myocardial infarction, one inpatient diagnosis during the previous year	Yes versus no	1.224	1.197	1.251	<0.0001
Hospital admission	Yes versus no	1.137	1.126	1.148	<0.0001

Supplementary Table S12. Odds ratios for hospitalizations for ambulatory care-sensitive conditions in CHD patients, based on generalized linear models of the longitudinal analysis from 2011-2018
CHD, coronary heart disease; CHF, chronic heart failure; COPD, chronic obstructive pulmonary disease; DMP, disease management program; DM, diabetes mellitus.

Variable	Interpretation	Odds Ratio	Lower 95% CI	Upper 95% CI	p-value
Intervention effect at the start of the study	Primary care program versus control group	0.970	0.936	1.005	0.0969
Time trend in the unexposed group	Per year	0.983	0.977	0.989	<0.0001
Time trend modified by intervention	Primary care program versus control group per year	0.984	0.976	0.992	<0.0001
Age	In decades	1.010	0.998	1.021	0.0951
Sex	Male versus female	0.849	0.831	0.867	<0.0001
Group practice	Yes versus no	1.005	0.985	1.025	0.6222
DMP DM	Yes versus no	0.965	0.944	0.986	0.0015
DMP CHD	Yes versus no	0.807	0.790	0.824	<0.0001
Rural practice	No versus yes	1.016	0.996	1.036	0.1141
Charlson Comorbidity Index	Per point	1.192	1.188	1.197	<0.0001
Need for nursing	Yes versus no	1.793	1.752	1.835	<0.0001
Renal failure	Yes versus no	1.594	1.559	1.630	<0.0001
CHF	Yes versus no	0.969	0.950	0.989	0.0027
Hypertension	Yes versus no	1.600	1.554	1.648	<0.0001
COPD	Yes versus no	2.037	1.992	2.084	<0.0001
Atrial fibrillation	Yes versus no	0.995	0.972	1.018	0.6446
Myocardial infarction, one inpatient diagnosis during the previous year	Yes versus no	1.075	1.034	1.118	0.0003
Myocardial infarction as main inpatient diagnosis	Yes versus no	1.150	1.089	1.215	<0.0001
Hospital admission	Yes versus no	1.514	1.485	1.543	<0.0001

Supplementary Table S13. Odds ratios for cardiovascular-related hospital admissions in CHD patients, based on generalized linear models of the longitudinal analysis from 2011-2018

CHD, coronary heart disease; CHF, chronic heart failure; COPD, chronic obstructive pulmonary disease; DMP, disease management program; DM, diabetes mellitus.

Variable	Interpretation	Odds Ratio	Lower 95% CI	Upper 95% CI	p-value
Intervention effect at the start of the study	Primary care program versus control group	0.921	0.901	0.942	<0.0001
Time trend in the unexposed group	Per year	0.965	0.961	0.969	<0.0001
Time trend modified by intervention	Primary care program versus control group per year	0.978	0.973	0.983	<0.0001
Age	In decades	0.963	0.956	0.969	<0.0001
Sex	Male versus female	1.010	0.997	1.024	0.1330
Group practice	Yes versus no	1.005	0.993	1.018	0.3972
DMP DM	Yes versus no	0.655	0.646	0.665	<0.0001
DMP CHD	Yes versus no	1.109	1.095	1.124	<0.0001
Rural practice	No versus yes	1.009	0.997	1.022	0.1476
Charlson Comorbidity Index	Per point	1.260	1.257	1.263	<0.0001
Need for nursing	Yes versus no	0.996	0.980	1.012	0.5957
Renal failure	Yes versus no	1.645	1.621	1.670	<0.0001
CHF	Yes versus no	1.046	1.033	1.060	<0.0001
Hypertension	Yes versus no	1.780	1.749	1.813	<0.0001
COPD	Yes versus no	0.969	0.954	0.984	0.0001
Atrial fibrillation	Yes versus no	1.399	1.378	1.420	<0.0001
Myocardial infarction, one inpatient diagnosis during the previous year	Yes versus no	1.458	1.421	1.495	<0.0001
Hospital admission	Yes versus no	1.199	1.185	1.214	<0.0001

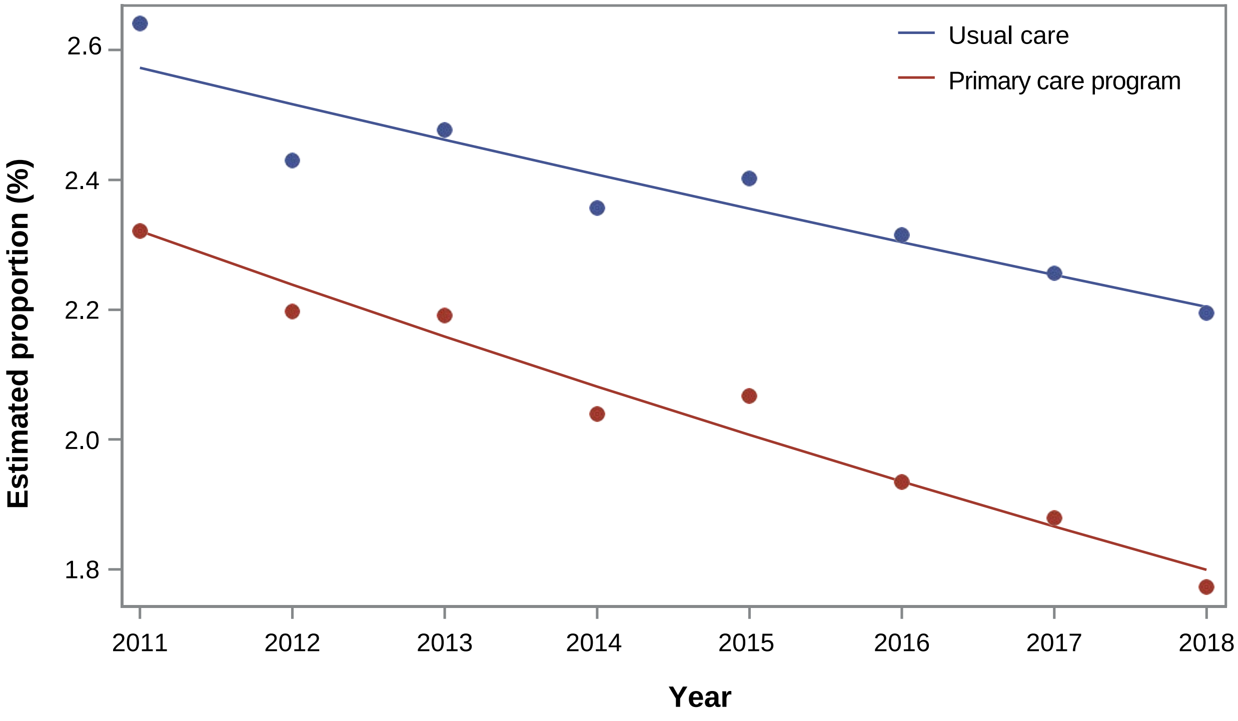

Supplementary Figure S1. Estimated proportions of hospitalizations for ambulatory care-sensitive conditions in the elderly, based on generalized linear models of the longitudinal analysis from 2011-2018

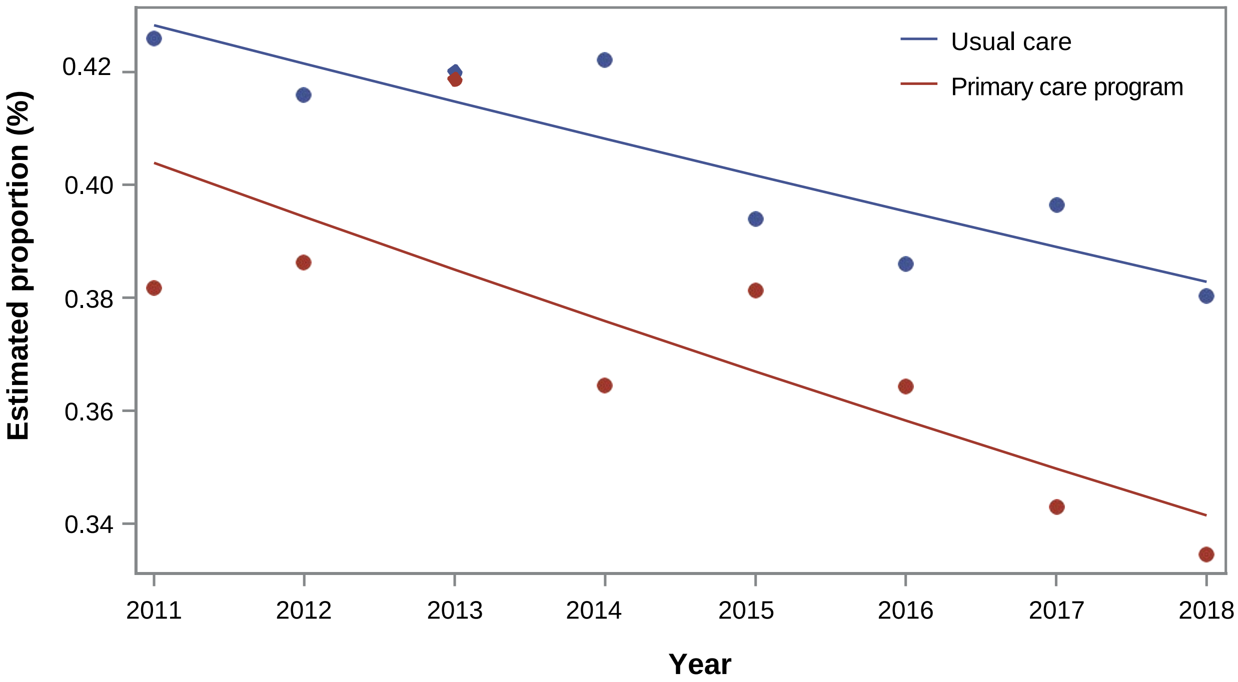

Supplementary Figure S2. Estimated proportions of hospitalization for hip fracture in the elderly, based on generalized linear models of the longitudinal analysis from 2011-2018

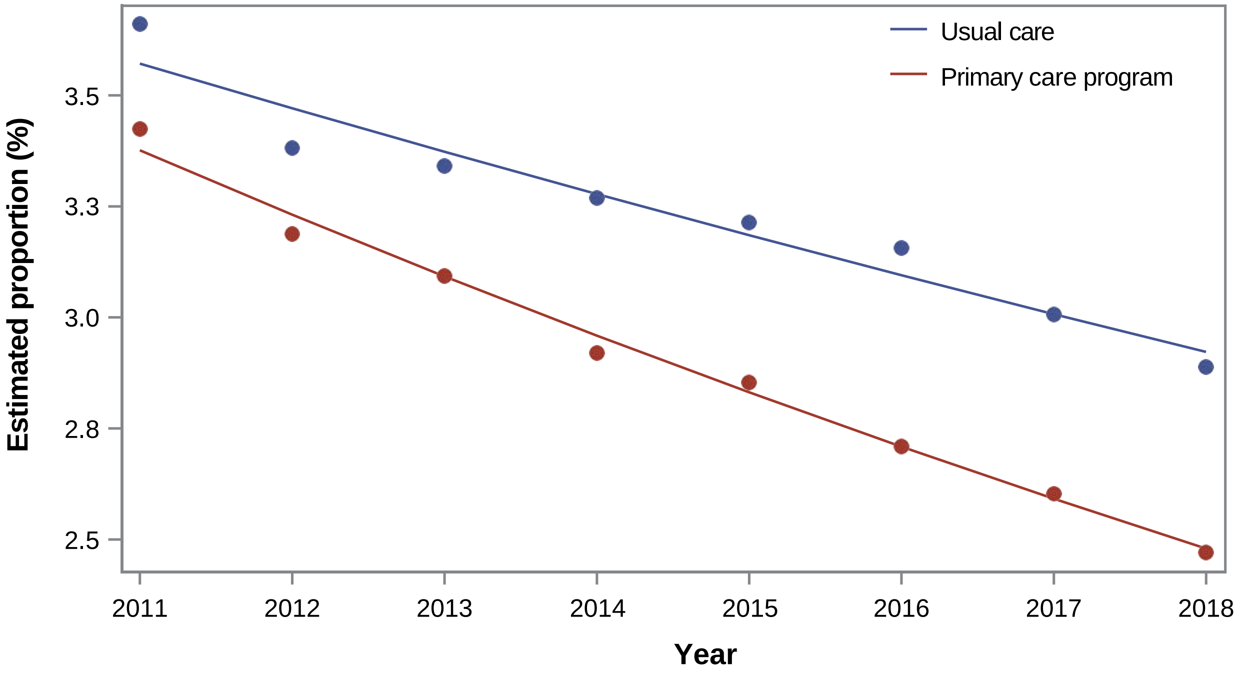

Supplementary Figure S3. Estimated proportions of ambulatory care-sensitive conditions in diabetics, based on generalized linear models of the longitudinal analysis from 2011-2018

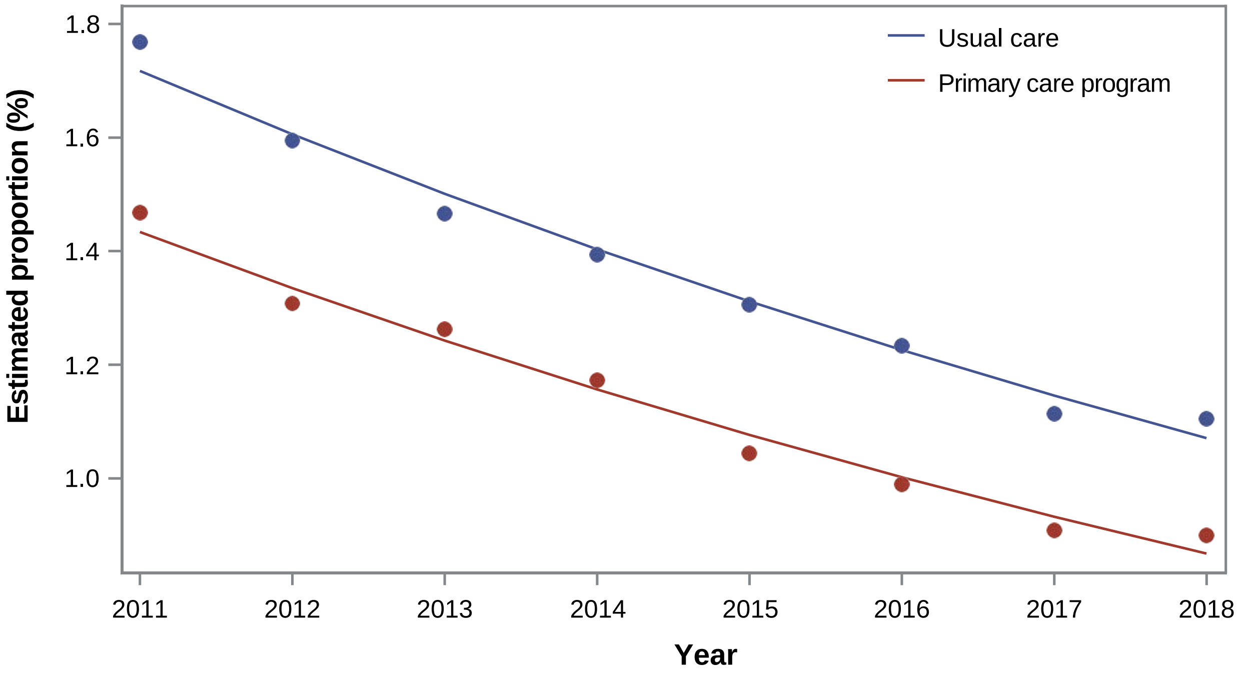

Supplementary Figure S4. Estimated proportions of diabetic-related hospital admissions in diabetics, based on generalized linear models of the longitudinal analysis from 2011-2018

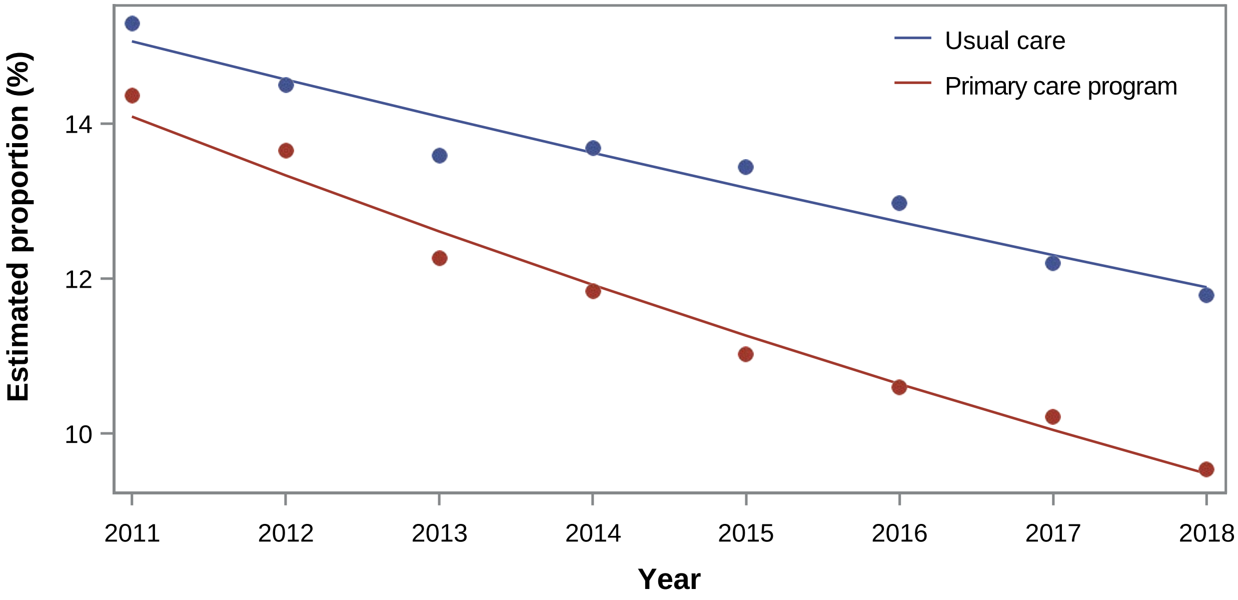

Supplementary Figure S5. Estimated proportions of cardiovascular-related hospital admissions in CHF patients, based on generalized linear models of the longitudinal analysis from 2011-2018

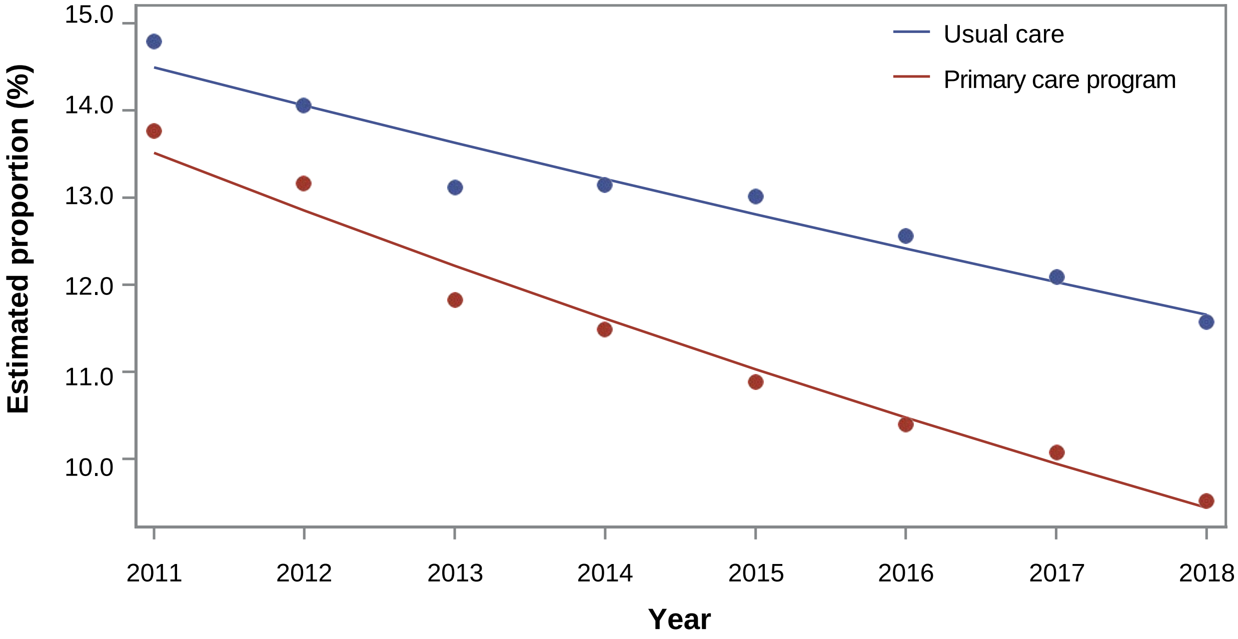

Supplementary Figure S6. Estimated proportions of cardiovascular-related hospital admissions in CHD patients, based on generalized linear models of the longitudinal analysis from 2011-2018

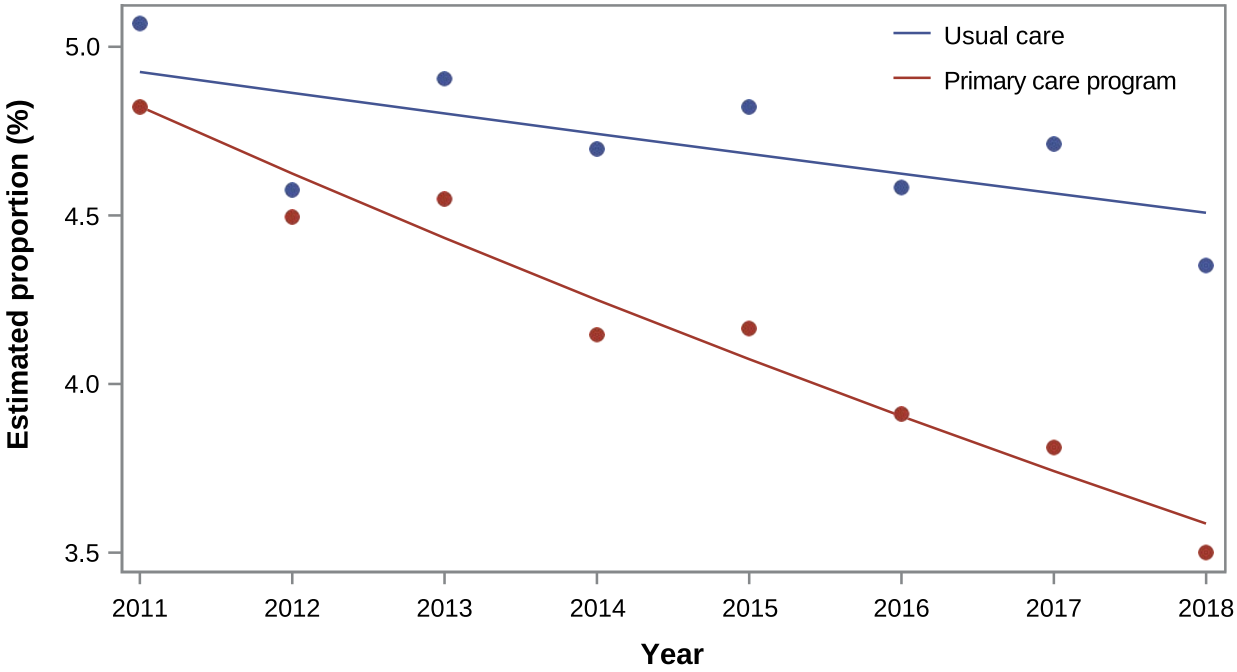

Supplementary Figure S7. Estimated proportions of hospitalizations for ambulatory care-sensitive conditions in CHF patients, based on generalized linear models of the longitudinal analysis from 2011-2018

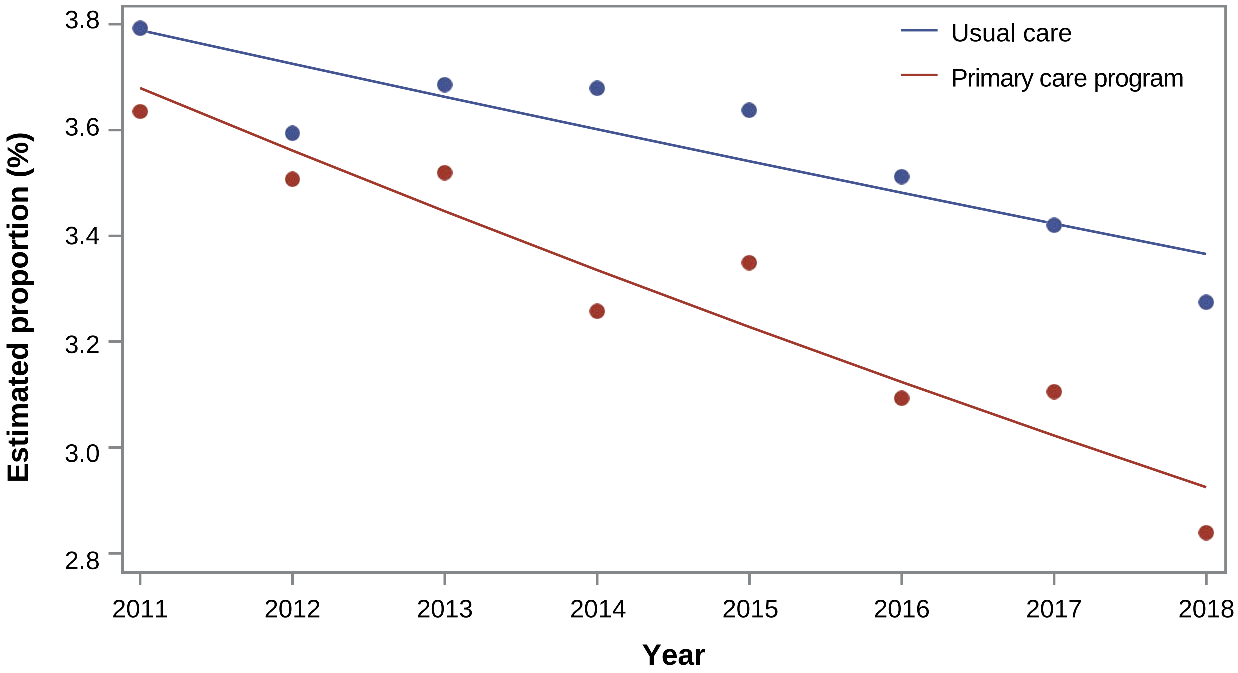

Supplementary Figure S8. Estimated proportions of hospitalizations for ambulatory care-sensitive conditions in CHD patients, based on generalized linear models of the longitudinal analysis from 2011-2018

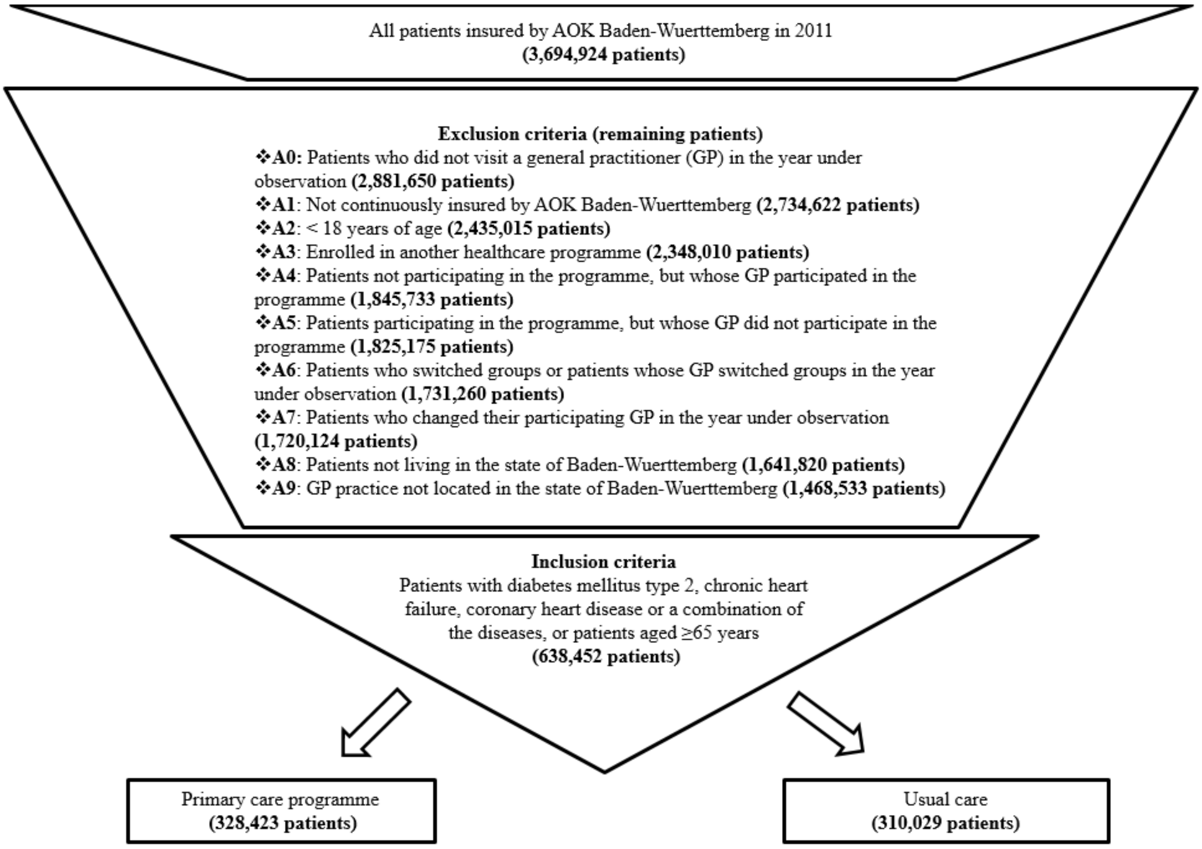

Supplementary Figure S9. Exemplary flow chart for 2011 showing general inclusion and exclusion criteria
All inclusion and exclusion criteria were applied annually.
